# New Memoirs of the Academy of Dijon, Relative to the Sciences and Arts

**Published:** 1783

**Authors:** 


VI. *Nouveaux Memoires de l'Academie de Dijon, pour la partie des Sciences et Arts.* i. e. *New Memoirs of the Academy of Dijon, relative to the Sciences and Arts.* Vol. I. for 1782. Part I. 8vo. Dijon 1783. 255 pages.

THE plan of this new work has already been announced to our readers *. The part now before us contains eleven eſſays, viz. 1. *Inquiries relative to the improvement of the preparation of colours employed in painting. By* M. de Morveau.—2. *An eſſay on the conſtrućtion of hoſpitals, written with a view to aſcertain the*

* See p. 99.

*best method of keeping the air of infirmaries pure and wholesome. By* M. Maret.—As a proof that air vitiated by respiration is specifically heavier than pure atmospheric air, M. Maret observes, that in the Hotel Dieu at Lyons, several birds were suspended in a cage from the dome of one of the wards, the air of which was deemed very unhealthy, and after having been kept there for the space of a fortnight, seemed to be in perfect health. Fresh meat in a similar situation, we are told, retained its sweetness upwards of five days, but became putrid in twenty-four hours when suspended in a lower part of the ward near the beds of the patients. He offers a theory of ventilation which will perhaps not be readily admitted. He supposes that the current of air passing into an apartment is composed of rays which converge to the point at which they enter, and from thence diverge at angles, more or less acute, according to their number and rapidity. M. Maret goes so far as to determine the form, direction, and effects of the cones that result from this divergent course of the rays. He applies this theory to the wards of hospitals, which he advises to be constructed of an elliptical form, with a door or window at each extremity.

tremity. The upper part of the walls and the roof are to be of the fame fhape.—3. *Obfervations on the congelation of concentrated vitriolic acid. By* M. de Morveau.—4. *Aftronomical obfervations. By* M. Roger.—5. *An account of the ores of copper called mountain green* (verd de montagne) *and mountain blue* (bleu de montagne) *By* M. de Morveau.—6. *On the air obtained from cream of lime and minium. By* M. Maret.—The experiments related in this effay prove that the air procured from cream of lime is fixed air, while that obtained from minium is dephlogifticated air mixed with about a fixth part of fixed air.—7. *Obfervations on the fluices of navigable canals. By* M. Gauthey.—8. *Mineralogical and chemical obfervations on heavy fpar, and the manner of procuring from it that kind of earth which by the French is called* Barote *or* terre barotique. *By* M. de Morveau.—9. *Remarks on the omphalo-mefenteric veffels. By* M. Chauffier.—We have here an account of two fmall arteries which pafs from the umbilicus to the mefentery, and which the author thinks have not been before noticed by anatomical writers.—10. *Remarks on biliary concretions, and on the efficacy of a mixture of vitriolic æther and fpirit of turpentine in the colic produced by thofe concretions. By* M. Durande.

In

—In this paper the author defcribes the fymptoms of the colic in queftion, and the different remedies which have hitherto been employed againft it. His account of the cafes in which the æther and fpirit of turpentine have been adminiftered, is referved for the fecond part of the volume.—11. *Meteorological Hiftory of* 1781. *By* M. Maret.

